# Nucleophilic Arylation of Halopurines Facilitated by Brønsted Acid in Fluoroalcohol

**DOI:** 10.3390/molecules24213812

**Published:** 2019-10-23

**Authors:** Naoko Takenaga, Toshitaka Shoji, Takayuki Menjo, Akiko Hirai, Shohei Ueda, Kotaro Kikushima, Tomonori Hanasaki, Toshifumi Dohi

**Affiliations:** 1 Faculty of Pharmacy, Meijo University, 150 Yagotoyama, Tempaku-ku, Nagoya 468-8503, Japan; 2 College of Pharmaceutical Sciences, Ritsumeikan University, 1-1-1 Nojihigashi, Kusatsu, Shiga 525–8577, Japan; ph0088sh@ed.ritsumei.ac.jp (T.S.); akiko.xp@gmail.com (A.H.); kixy@fc.ritsumei.ac.jp (K.K.); 3 Department of Applied Chemistry, College of Life Sciences, Ritsumeikan University, 1-1-1 Nojihigashi, Kusatsu, Shiga 525–8577, Japan; sb0067xs@ed.ritsumei.ac.jp (T.M.); sc0029ev@ed.ritsumei.ac.jp (S.U.); hanasaki@sk.ritsumei.ac.jp (T.H.)

**Keywords:** purine, nucleobase, aromatic substitution, arylation, fluoroalcohol

## Abstract

Various aryl-substituted purine derivatives were synthesized through the direct arylation of halopurines with aromatic compounds, facilitated by the combination of triflic acid and fluoroalcohol. This metal-free method is complementary to conventional coupling reactions using metal catalysts and reagents for the syntheses of aryl-substituted purine analogues.

## 1. Introduction

Biogenic purine bases are heteroaromatic compounds that constitute the basic subunits of DNA and RNA and play a crucial role in biological processes. In addition to these natural nucleosides, various chemically modified purine nucleosides have recently been discovered, and detailed analyses of their bioactivities have attracted much attention. Purine derivatives bearing an aryl substituent are of particular interest among these extensively studied classes of compounds, and their preparation has gained much attention owing to the promising biological properties of the derivatives, such as cytotoxicity and antitumor activity [1,2,3]. In addition, their applications as biological probes have also been consistent with the synthetic advances in artificial purine compounds [4].

**Scheme 1:**
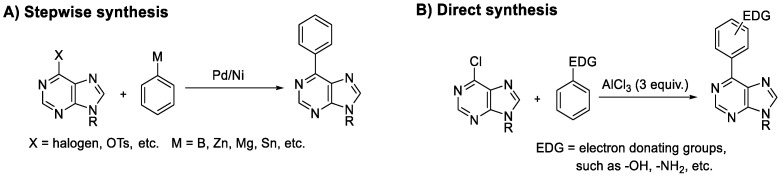
General synthetic routes to obtain aryl-substituted purines from halopurines. (**A**) Stepwise synthesis. (**B**) Direct synthesis.

The classical methods for the preparation of purines bearing aryl substituents are based on heterocyclization; however, the cyclization methodology usually requires multistep procedures. Consequently, the synthesis of the target aryl purines afforded only moderate to low yields [5]. The recent methods for the synthesis of aryl-substituted purines involve the transition metal-catalyzed cross-coupling reactions of aryl organometallics (Ar-M) with halopurines (Scheme 1A) [6,7]. For example, Suzuki–Miyaura [8,9,10,11,12,13,14,15,16], Stille [17,18], Negishi [19], and Kumada [20] coupling reactions have been frequently used for the preparation of aryl-substituted purines. Indeed, these approaches represent versatile and reliable synthetic methods; however, these coupling reactions require stoichiometric amounts of metallic reagents and the protection of the nucleophilic functional groups—such as the hydroxyl and amino groups—in the substrates. Hence, direct arylation of 6-chloropurines by electron-rich arenes using a three-fold excess of aluminum chloride (AlCl_3_) was reported by Guo’s group as an alternative method for preparing aryl purines in a short synthetic step (Scheme 1B) [21].

**Scheme 2:**
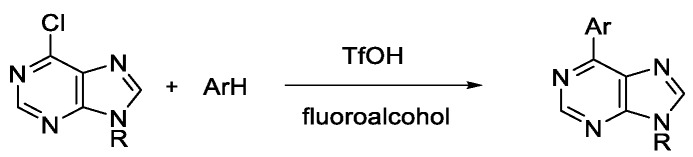
Direct arylation of halopurines facilitated by Brønsted acid in fluoroalcohol. ArH: aryl nucleophile, TfOH: triflic acid.

Despite these synthetic advances brought about by the alternative method, there unfortunately remain some limitations regarding the structural diversity of the obtainable aryl purines. To the best of our knowledge, the preparation of *N*-7-substituted 6-arylpurines has seldom been reported in the scientific literature [22,23,24]. To expand the synthetic scope for obtaining highly functionalized aryl purines with greater structural and steric diversities, developing a new practical method for preparing a variety of aryl-substituted purines is still necessary. In our continuous study on the development of a new method for the synthesis of functionalized nucleobases [25,26,27], we would like to report herein the metal-free arylation of purine derivatives facilitated by the combination of triflic acid and fluoroalcohol (Scheme 2).

## 2. Results and Discussions

Fluoroalcohols, such as 1,1,1,3,3,3-hexafluoro-2-propanol (HFIP) and 2,2,2-trifluoroethanol (TFE), possess specific properties that differentiate them from other non-fluorinated alcohols; they are highly polar [28] and weakly nucleophilic [29] and exhibit remarkable hydrogen-bond donor abilities [30]. Owing to their unique physical properties, these fluoroalcohols can dramatically direct the course of reactions; thus, as a means of developing new reactions, the authors utilized HFIP and TFE as attractive and distinctive alternatives to ordinary solvents in hypervalent iodine chemistry [31,32,33]. In these studies, we unexpectedly discovered the metal-free S_N_Ar-type arylation of heteroaromatic diaryliodonium salts by nucleophilic aromatic compounds facilitated by Lewis and Brønsted acids, i.e., boron trifluoride, trimethylsilyl triflate, and triflic acid (TfOH), in fluoroalcohols [34,35,36]. Golding’s group also reported that the combination of trifluoroacetic acid (TFA) and TFE allowed the amination of halopurines by various substituted anilines under metal-free conditions; this method involves C–N bond formation [37,38,39,40]. Meanwhile, the metal-free nucleophilic arylation of halopurines involving C–C bond formation has not been reported.

In a pilot experiment, we first examined the S_N_Ar-type coupling reaction of 6-chloropurine **1a** initiated by a Brønsted acid [34,35], using methyl indole **2a** as an aromatic nucleophile in HFIP as the model case (Table 1). In order to optimize the coupling reaction, varying equivalents (Entries 1–4) of TfOH were used, and the desired arylation product of purine **3aa** was obtained in excellent yield when we used 0.5 to 1.0 equiv. of TfOH for the reactions (Entries 3 and 4). The usage of alternative Brønsted acids as additives, such as H_3_PO_4_, *p*-TsOH, and even TFA [37,38,39,40], was not as effective and provided inferior results in comparison with the use of TfOH. The fluoroalcohol HFIP plays an essential role in the reaction, and a solvent mixture of HFIP and 1,2-dichloroethane (DCE) did not smoothly produce the coupling product **3aa**. (Entry 5). Also, the replacement of HFIP with TFE and the use of methanol and acetonitrile as the solvent instead of HFIP yielded low or null amounts of product **3aa**.

To evaluate the generality of the reaction system, the substrate scope under the optimized reaction conditions was examined (Table 2). The reaction of *N*-protected 9-benzyl-6-chloro-9*H*-purine **1b** cleanly favored the corresponding product **3ba** with good yield. When non-*N*-protected indoles **2b**–**d** were subjected to analogous reaction conditions, the desired products **3bb**–**bd** were also obtained in excellent yields. However, it was revealed that the reaction of indoles bearing electron-withdrawing groups, such as 5-nitroindole **2e**, did not proceed under these reaction conditions due to the deactivation of the aromatic nucleophile by hydrogen bonding with HFIP [41,42,43]. Furthermore, other electron-rich arenes were as compatible as the aromatic nucleophiles; similarly, good results were obtained from the coupling reactions with 1-naphthol **2f**, 1-methoxynaphthalene **2g**, several alkoxybenzenes **2h**,**i**, and resorcinol **2j**.

**Scheme 3:**
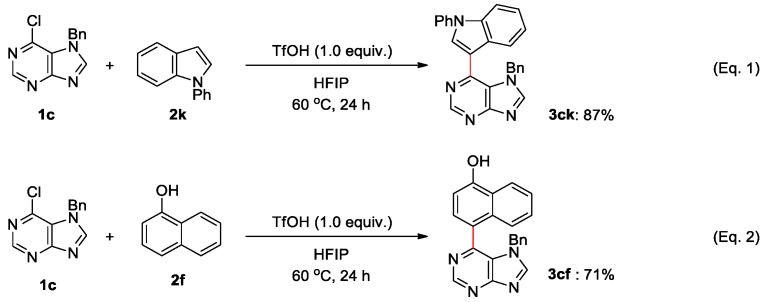
Synthesis of 7-substituted 6-aryl purines.

One of the significant advantages of the present reaction system is the production of structural and sterically diverse *N*-7-substituted 6-arylpurines; these arylpurines are not easily accessible by other synthetic methods [22,23,24]. As a result, the proposed reaction conditions were also utilized for the coupling of 7-benzyl-6-chloro-7*H*-purine **1c** with indole **2k** and naphthalene nucleophile **2f** to afford the corresponding 6-arylated *N*-7-substituted purines **3ck** and **3cf** in good yields (Scheme 3).

**Scheme 4:**
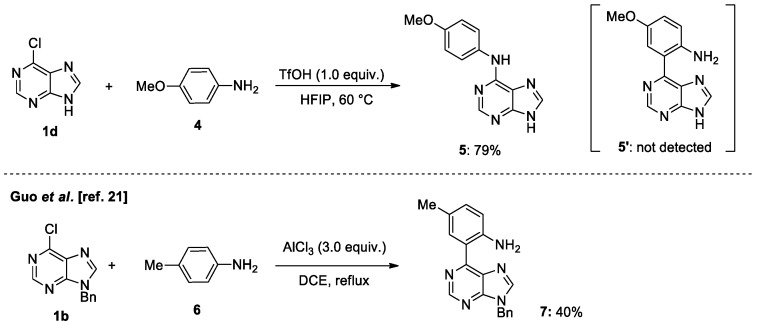
Formation of different products using Brønsted acid and aluminum chloride as activators.

When using *p*-anisidine **4** as a substrate, our reaction system with TfOH became valuable for the chemoselective *N*-arylation of halopurines at the 6 position under mild temperature (Scheme 4) [37]. We subjected 9*H*-chloropurine **1d** and aniline **4** to our optimized conditions at 60 °C, obtaining selective *N*-arylation that smoothly provided *N*-(4-methoxyphenyl)-9*H*-purine-6-amine **5** in 79% yield, without the formation of the C-arylated purine coupling product **5′**. On the other hand, Guo’s group previously reported the reaction of purines and anilines or naphthylamines in the presence of a three-fold excess of AlCl_3_ in DCE, which alternatively gave the C-arylated coupling products and likewise the biaryl **5′** [21]. Therefore, our reaction system is complementary to the AlCl_3_-mediated coupling reaction [21] for the syntheses of C6-aryl-substituted purine derivatives in view of product selectivity.

The success of the metal-free coupling reaction relies on the use of HFIP as the solvent. Although the precise role of HFIP [44,45,46] remains unclear, we presume that HFIP can increase the acidity of TfOH (Brønsted acid activation by H-bond donor) to enhance the reactivity of halopurine electrophiles through the purine nitrogen atoms [47,48,49,50]. Importantly, HFIP offers a means of improving the leaving group ability of the chloride atom in the purine substrates through hydrogen bonding as well as solvation [51]. Recently, such unique role of fluoroalcohol as the H-bond donor has been discussed in several Brønsted acid catalyzed reactions in regard to its ability to accelerate substitution processes [37,47,48,49,50,51]. Interestingly, these cases would involve intermediates activated by hydrogen bonding with fluoroalcohol, and, with our present system, the formation of a similar intermediate would also be expected to facilitate the aromatic substitution reactions.

## 3. Conclusions

In conclusion, we have developed a new metal-free coupling method of halopurines for the syntheses for diverse C6-aryl-substituted purine derivatives based on Brønsted acid activation. The combination of TfOH and HFIP is an efficient and practical methodology for the direct nucleophilic arylation of halopurines under mild conditions. We have elucidated that the unique properties of HFIP (hydrogen-bonding formation and weak nucleophilicity) could facilitate the direct arylation of halopurines by various nucleophilic arene molecules. Further investigations on the utilization of the obtained purine biaryls are currently underway in our research group.

## 4. Experimental Section

The melting points (mp) are uncorrected. The ^1^H-NMR (and ^13^C-NMR) spectra of the coupling products **3** and **5** were recorded by a JEOL JMN-400 spectrometer (JEOL Ltd., Tokyo, Japan) operating at 400 MHz (100 MHz for ^13^C-NMR) in DMSO-*d*_6_ at 25 °C with tetramethylsilane as the internal standard. The data are reported as follows: chemical shift in part per million (δ), multiplicity (s = singlet, d = doublet, t = triplet, q = quartet, br = broad singlet, m = multiplet), integration, and coupling constant (Hz). The infrared spectra (IR) were obtained using a Hitachi 270–50 spectrometer (Hitachi Ltd., Tokyo, Japan); absorptions are reported in reciprocal centimeters (cm^−1^) for representative peaks. High-resolution mass spectra were measured with a Thermo Scientific Exactive Plus Orbitrap (Thermo Fisher Scientific., Inc., Waltham, MA, USA). All chemicals used in this study are commercially available and were used without further purification. Regarding fluoroalcohol, we used commercial water-containing hexafluoroisopropanol (HFIP) as supplied for the reactions.

### 4.1. General Procedure for Brønsted Acid Catalyzed Arylation of Halopurines in Fluoroalcohol (Table 2 and Scheme 3)

To a stirred solution of chloropurine **1** (0.50 mmol) in hexafluoroisopropanol (5 mL), aromatic nucleophile **2** (0.55 mmol, 1.1 equiv) and trifluoromethanesulfonic acid (TfOH, 44 μL, 0.5 mmol, 1 equiv) were successively added. The resulting mixture was stirred at 60 °C for 24 h. After completion of the reaction checked by TLC, the reaction mixture was poured into sat. NaHCO_3_ aqueous. The resultant solution was extracted with ethyl acetate, dried with solid sodium sulfate, and then concentrated. The residue was purified by short-column chromatography on silica gel using hexane-ethyl acetate as the eluent to give the purine aromatic-linked compound **3** in the indicated yield in Table 2 or Scheme 3.

*Compound 6-(1-methyl-1H-indol-3-yl)-7H-purine* (**3aa**). A yellow powder, mp 346–350 °C. IR: 3647, 1732 cm^−1^. ^1^H-NMR (400 MHz, DMSO-*d*_6_) δ 3.95 (s, 3H), 7.21–7.31 (m, 2H), 7.55 (d, *J* = 7.9 Hz, 1H), 8.49 (s, 1H), 8.81–8.84 (m, 2H), 8.97 (s, 1H), 13.4 (bs, 1H) ppm; ^13^C-NMR (100 MHz, DMSO-*d*_6_) δ 33.1, 110.4 (x 2), 121.0, 122.4, 122.9, 126.2, 128.0, 136.2, 137.2, 142.7, 151.3, 152.1, 152.3 ppm; HRMS (DART): Calcd. for C_14_H_12_N_5_ [M + H]^+^: 250.1087, found: 250.1087.

*Compound 6-(1-methyl-1H-indol-3-yl)-9-phenylmethyl)-9H-purine* (**3ba**) [21]. A yellow powder, mp 163–166 °C. IR: 3047, 2932, 1581, 1536, 1498, 1475 cm^−1^. ^1^H-NMR (400 MHz, DMSO-*d*_6_) δ 3.96 (s, 3H), 5.51(s, 2H), 7.23–7.59 (m, 7H), 7.57 (d, 1H, *J* = 7.8 Hz), 8.68 (s, 1H), 8.80 (d, 1H, *J* = 7.8 Hz), 8.86 (s, 1H), 8.96 (s, 1H) ppm; ^13^C-NMR (100 MHz, DMSO-*d*_6_) δ 33.0, 54.9, 110.2, 110.3, 121.1, 122.5, 122.8, 126.2, 127.6, 127.8, 128.1, 128.7, 136.3, 136.8, 137.2, 144.4, 150.3, 152.3, 152.7 ppm.

*Compound 6-(1H-indol-3-yl)-9-phenylmethyl-9H-purine* (**3bb**) [21]. A yellow powder, mp 178–180 °C. IR: 3631, 1688 cm^−1^. ^1^H-NMR (400 MHz, DMSO-*d*_6_) δ 5.51 (s, 2H), 7.17–7.41 (m, 7H), 7.52 (d, 1H, *J* = 6.8 Hz), 8.66 (s, 1H), 8.76–8.89 (m, 2H), 8.99 (s, 1H), 12.0 (s, 1H) ppm; ^13^C-NMR (100 MHz, DMSO-*d*_6_) δ 46.3, 111.2, 112.0, 120.9, 122.4, 122.7, 125.7, 127.9, 128.4, 128.8, 132.8, 136.6, 136.9, 144.5, 150.3, 152.3, 153.1 ppm.

*Compound 6-(5-methyl-1H-indol-3-yl)-9-phenylmethyl-9H-purine* (**3bc**). A brown liquid, IR: 3649, 1690, 1559, 1540 cm^–1^. ^1^H-NMR (400 MHz, DMSO-*d*_6_) δ 2.36 (s, 3H), 5.45 (s, 2H), 7.08 (d, *J* = 8.3 Hz, 1H), 7.26−7.36 (m, 6H), 8.53 (s, 1H), 8.59 (s, 1H), 8.82 (s, 1H), 8.90 (s, 1H), 11.8 (s, 1H) ppm; ^13^C-NMR (100 MHz, DMSO-*d*_6_) δ 21.5, 46.0, 110.8, 111.7, 122.3, 124.0, 126.0, 127.6, 127.9, 128.3, 128.8, 129.5, 132.9, 135.0, 136.9, 144.3, 150.2, 152.3, 153.2 ppm; HRMS (DART): Calcd. for C_21_H_18_N_5_^+^ [M + H]^+^: 340.1557, found: 340.1557.

*Compound 6-(5-methoxy-1H-indol-3-yl)-9-phenylmethyl-9H-purine* (**3bd**). A brown solid, mp 229–231 °C. IR: 3595, 1704, 1559, 1508, 1437 cm^−1^. ^1^H-NMR (400 MHz, DMSO-*d*_6_) δ 3.89 (s, 3H), 5.57 (s, 2H), 6.92 (d, 1H, *J* = 8.6 Hz), 7.31–7.49 (m, 6H), 8.38 (s, 1H), 8.71 (s, 1H), 8.93 (s, 1H), 9.01 (s, 1H), 11.9 (s, 1H) ppm; ^13^C-NMR (100 MHz, DMSO-*d*_6_) δ 46.3, 55.4, 104.6, 111.0, 112.2, 112.7, 126.4, 127.6, 127.9, 128.2, 128.8, 131.6, 133.2, 136.9, 144.3, 150.2, 152.3, 153.2, 154.7 ppm; HRMS (DART): Calcd. for C_21_H_18_N_5_O^+^ [M + H]^+^: 356.1506, found: 356.1507.

*Compound 4-(7H-purin-6-yl)-naphthalene-1-ol* (**3af**). A yellow powder, mp 204–208 °C. IR: 3650, 1541 cm^−1^. ^1^H-NMR (400 MHz, DMSO-*d*_6_) δ 7.09 (d, 1H, *J* = 8.6 Hz), 7.51–7.57 (m, 2H), 8.06–8.63 (m, 4H), 9.06 (s, 1H), 10.8 (bs, 1H), 13.3 (bs, 1H) ppm; ^13^C-NMR (100 MHz, DMSO-*d*_6_) δ 107.8, 122.6, 123.5, 125.0, 125.3, 125.8, 127.3, 131.4, 132.2, 152.0, 155.5 ppm; HRMS (DART): Calcd. for C_15_H_11_N_4_O^+^ [M + H]^+^: 263.0927, found: 263.0928.

*Compound 6-(4-methoxynaphthalen-1-yl)-7H-purine* (**3ag**). A yellow powder, mp 170–172 °C. IR: 3629, 1704, 1542, 1508 cm^–1^. ^1^H-NMR (400 MHz, DMSO-*d*_6_) δ 4.06 (s, 3H), 7.16 (d, 1H, *J* = 8.6 Hz), 7.49–7.59 (m, 2H), 8.22–8.30 (m, 3H), 8.58 (bs, 1H), 9.02 (s, 1H), 13.6 (bs, 1H) ppm; ^13^C-NMR (100 MHz, DMSO-*d*_6_) δ 56.0, 103.9, 121.7, 124.8, 125.0, 125.6, 127.1, 131.5, 151.6, 156.3 ppm; HRMS (DART): Calcd. for C_16_H_13_N_4_O^+^ [M + H]^+^: 277.1084, found: 277.1082.

*Compound 6-(4-methoxynaphthalen-1-yl)-9-phenylmethyl-9H-purine* (**3bg**) [21]. A white solid, mp 197–198 °C. IR: 3672, 2968, 1507 cm^−1^. ^1^H-NMR (400 MHz, DMSO-*d*_6_) δ 4.02 (s, 3H), 5.55 (s, 2H), 7.12 (d, 1H, *J* = 8.0 Hz), 7.24–7.36 (m, 3H), 7.42 (d, 2H, *J* = 7.4 Hz), 7.48–7.55 (m, 2H), 8.13 (d, 1H, *J* = 8.6 Hz), 8.24–8.28 (m, 1H), 8.44–8.49 (m, 1H), 8.75 (s, 1H), 9.09 (s, 1H) ppm; ^13^C-NMR (100 MHz, DMSO-*d*_6_) δ 46.5, 55.8, 103.8, 121.7, 124.5, 125.0, 125.4, 125.9, 127.0, 127.8, 127.9, 128.7, 131.6, 131.8, 136.5, 146.1, 151.7, 151.8, 156.4, 156.5 ppm.

*Compound 6-(1,3,5-trimethoxyphen-4-yl)-9-phenylmethyl-9H-purine* (**3bh**) [21]. A white solid, mp 251–253 °C. IR: 3650, 1698 cm^−1^. ^1^H-NMR (400 MHz, DMSO-*d*_6_) δ 3.58 (s, 6H), 3.84 (s, 3H), 5.49 (s, 2H), 6.35 (s, 2H), 7.27–7.47 (m, 5H), 8.61 (s, 1H), 8.90 (s, 1H) ppm; ^13^C-NMR (100 MHz, DMSO-*d*_6_) δ 46.6, 55.5, 55.7, 91.0, 106.6, 128.1, 128.8, 133.6, 136.6, 145.7, 150.8, 151.8, 154.2, 158.7, 162.0 ppm.

*Compound 6-(1,3-dimethoxyphen-4-yl)-9-phenylmethyl-9H-purine* (**3bi**) [52]. A white solid, mp 125–127 °C. IR: 3671, 1707 cm^−1^. ^1^H-NMR (400 MHz, DMSO-*d*_6_) δ 3.74 (s, 3H), 3.83 (s, 3H), 5.50 (s, 2H), 6.66 (d, 1H, *J* = 8.8 Hz), 6.73 (s, 1H), 7.24–7.42 (m, 5H), 7.53 (d, 1H, *J* = 8.3 Hz), 8.66 (s, 1H), 8.93 (s, 1H) ppm; ^13^C-NMR (100 MHz, DMSO-*d*_6_) δ 46.5, 55.4, 55.7, 98.9, 105.2, 117.8, 127.8, 128.0, 128.8, 131.9, 132.5, 136.7, 145.6, 151.1, 151.8, 155.4, 158.8, 162.0 ppm.

*Compound 4-(9-phenylmethyl-9H-purin-6-yl)-benzene-1,3-diol* (**3bj**) [21]. A yellow solid, mp 250–253 °C. IR: 3691, 2983, 1686, 1507, 1318 cm^−1^. ^1^H-NMR (400 MHz, DMSO-*d*_6_) δ 5.47 (s, 2H), 6.36 (s, 1H), 6.48 (d, 1H, *J* = 8.8 Hz), 7.20–7.34 (m, 5H), 8.72 (s, 1H), 8.79 (s, 1H), 9.21 (d, 1H, *J* = 8.8 Hz), 14.6 (s, 1H) ppm; ^13^C-NMR (100 MHz, DMSO-*d*_6_) δ 46.6, 103.3, 106.4, 108.2, 109.1, 127.8, 128.8, 133.8, 136.5, 145.6, 149.5, 151.1, 154.4, 158.6, 162.5, 163.5 ppm.

*Compound 6-(1-phenyl-1H-indol-3-yl)-7-phenylmethyl-7H-purine* (**3ck**). A yellow powder, mp 191–194 °C. IR: 1693, 1521 cm^−1^. ^1^H-NMR (400 MHz, DMSO-*d*_6_) δ 5.54 (s, 2H), 7.26–7.39 (m, 7H), 7.49–7.74 (m, 6H), 8.72 (s, 1H), 8.91–8.95 (m, 2H), 9.14 (s, 1H) ppm; ^13^C-NMR (100 MHz, DMSO-*d*_6_) δ 46.4, 110.9, 112.8, 122.1, 123.2, 123.6, 124.5, 126.8, 127.6, 127.7, 127.9, 128.8, 130.1, 134.5, 136.0, 136.7, 138.2, 143.3, 145.0, 150.5, 152.0, 152.3 ppm. HRMS (DART): Calcd. for C_26_H_20_N_5_ [M + H]^+^: 402.1713, found: 402.1713.

*Compound 4-(7-phenylmethyl-7H-purin-6-yl)-naphthalene-1-ol* (**3cf**). A yellow powder, mp 259–263 °C. IR: 3613, 2980, 1697 cm^−1^. ^1^H-NMR (400 MHz, DMSO-*d*_6_) δ 4.96-5.04 (m, 2H), 6.18 (d, 2H, *J* = 7.3 Hz), 6.77 (t, 2H, *J* = 7.3 Hz), 6.86 (t, 1H, *J* = 7.3 Hz), 6.93 (d, 1H, *J* = 7.8 Hz), 7.18 (d, 1H, *J* = 8.3 Hz), 7.26 (t, 2H, *J* = 5.4 Hz), 7.42 (t, 1H, *J* = 7.3 Hz), 8.22 (d, 1H, *J* = 8.3 Hz), 8.91 (s, 1H), 9.04 (s, 1H), 10.7 (bs, 1H) ppm; ^13^C-NMR (100 MHz, DMSO-*d*_6_) δ 49.8, 107.0, 122.2, 123.4, 123.6, 124.3, 124.7, 124.9, 125.7, 126.9, 127.3, 127.9, 128.8, 132.3, 135.6, 150.8, 151.7, 152.0, 154.9, 161.6 ppm; HRMS (DART): Calcd. for C_22_H_17_N_4_O^+^ [M + H]^+^: 353.1397, found: 353.1395.

### 4.2. General Procedure for Brønsted Acid Catalyzed N-Coupling of Aniline Derivatives to Halopurines in Fluoroalcohol (Scheme 4)

To a stirred solution of 9*H*-chloropurine **1d** (77.3 mg, 0.50 mmol) in hexafluoroisopropanol (5 mL) *p*-methoxyaniline **4** (67.8 mg, 0.55 mmol, 1.1 equiv) and trifluoromethanesulfonic acid (TfOH, 44 μL, 0.5 mmol, 1 equiv) were successively added. The resulting mixture was stirred at 60 °C for 24 h. After completion of the reaction checked by TLC, the reaction mixture was poured into sat. NaHCO_3_ aqueous. The resultant solution was extracted with ethyl acetate, dried with solid sodium sulfate, and then concentrated. The residue was purified by short-column chromatography on silica gel using hexane-ethyl acetate as the eluent to give *N*-(4-methoxyphenyl)-9*H*-purine-6-amine **5** in 79% yield (95.3 mg, 0.395 mmol) as a white powder.

*CompoundN-(4-methoxyphenyl)-9H-purine-6-amine* (**5**) [53]. A white solid, mp 266–267 °C. IR: 3673, 3630 cm^−1^. ^1^H-NMR (400 MHz, DMSO-*d*_6_) δ 3.33 (s, 3H), 6.89 (d, 2H, *J* = 9.3 Hz), 7.79 (d, 2H, *J* = 8.8 Hz), 8.22 (s, 1H), 8.29 (s, 1H), 9.61 (s, 1H), 13.1 (bs, 1H) ppm; ^13^C-NMR (100 MHz, DMSO-*d*_6_) δ 55.2, 113.6, 119.2, 122.4, 132.8, 139.5, 150.2, 151.9, 154.9, 159.7 ppm.

## Figures and Tables

**Table 1:**
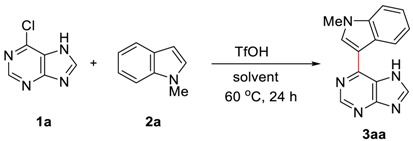
Optimization of the reaction conditions ^a^.

Entry	Solvent	TfOH	Yield (%) ^b^
1	HFIP	0.1 equiv.	8
2	HFIP	0.2 equiv.	54
3	HFIP	0.5 equiv.	89
4	HFIP	1.0 equiv.	quant.
5	HFIP/DCE = 9:1	1.0 equiv.	60

^a^ Reaction conditions: The reactions were performed using chloropurine **1a** (0.50 mmol), 1-methylindole **2a** (0.55 mmol), and TfOH in solvent (0.1 M). ^b^ Determined by ^1^H-NMR using nitromethane as an internal standard. HFIP: hexafluoroisopropanol, DCE: 1,2-dichloroethane.

**Table 2:**
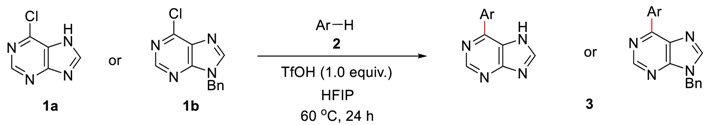
Scope of substrates ^a^.

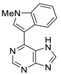	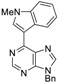	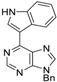	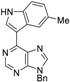
**3aa:** quant.	**3ba:** 82%	**3bb:** quant.	**3bc:** 96%
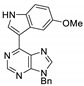	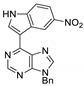	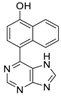	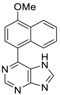
**3bd:** 90%	**3be:** n.r	**3af:** 97%	**3ag:** 85%
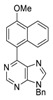	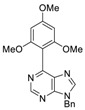	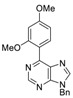	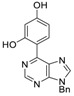
**3bg:** 92%	**3bh:** quant.	**3bi:** quant.	**3bj:** quant.

^a^ All the reactions were performed using chloropurine **1** (0.50 mmol), indole or aromatic nucleophile **2** (0.55 mmol), and TfOH (0.5 mmol, 1.0 equiv.) in HFIP (0.1 M) at 60 °C. The yields after isolation are indicated.
